# Supplementary material for: GpsB Coordinates Cell Division and Cell Surface Decoration by Wall Teichoic Acids in Staphylococcus aureus
Source: Microbiol Spectr. 2022 Jun 1;10(3):e01413-22. doi: 10.1128/spectrum.01413-22 (PMC9241681; doi:10.1128/spectrum.01413-22)
Supplement: Supplemental file 1 — Supplemental material. Download spectrum.01413-22-s0001.pdf, PDF file, 3.2 MB [file spectrum.01413-22-s0001.pdf]

Supplemental Information

GpsB coordinates cell division and cell surface decoration by wall teichoic acids in *Staphylococcus aureus*

Lauren R. Hammond[1^], Michael D. Sacco[2^], Sebastian J. Khan[1], Catherine Spanoudis[1], Abigail Hough[1], Yu Chen[2] and Prahathees J. Eswara[1*]

[1] Department of Cell Biology, Microbiology and Molecular Biology, University of South Florida, Tampa, Florida, USA

[2] Department of Molecular Medicine, University of South Florida, Tampa, Florida, USA

[^]LH and MS contributed equally. Author ordered determined by the seniority of involvement in this project.

[*]To whom correspondence should be addressed. Email: eswara@usf.edu

Supplemental Table and Figure Legends

Figure S1

(A) Stable production of *GpsB^{SA}-GFP was confirmed with western blot analysis of PY79 *B. subtilis* cells expressing *gpsB^{SA}-gfp* (GG8) or **gpsB^{SA}-gfp* (CS89-CS93, PE377, PE448) both with and without the addition of 1 mM IPTG. Clevage product is denoted by *. (B) The L35S *GpsB-GFP (PE448) mutation shows a clevage product detected via western blot (as denoted by *). GpsB^{SA}-GFP (GG8) and free GFP (RL4709) are shown for comparison. Blots probed with anti-GFP antibodies and anti-sigA for a loading control. (C) Localization of GpsB^{SA}-GFP (i) (GG8) and *GpsB^{SA}-GFP (ii – viii) (CS89-CS93, PE377, PE448) in *B. subtilis* PY79 cells. Images taken 3 h after the addition of 1 mM IPTG and membranes were visualized with SynaptoRed membrane dye. Scale bar is 1 µm.

Figure S2

Investigation of *GpsB dominant negative phenotypes. (A) Spot titer assay with *B. subtilis* PY79 cells co-expressing *gpsB^{SA}-gfp* (LH73) and one of the **gpsB^{SA}-gfp* intragenic suppressor mutations (LH75-LH80). Cultures were serially diluted and spotted onto plates in the absence (left) and presence (right) of 1 mM IPTG. (B) BACTH assay testing pairwise interactions of T18 - *GpsB^{SA} (LH43, LH45, LH47, LH49, LH51, LH53, LH55) with WT T25- GpsB^{SA} (LH40) and the negative (PE83/PE84; Neg) and positive (PE87/PE88; Pos) controls. Interactions on solid media with a dark pink color indicating a positive interaction. Image taken after 48 h of incubation (top). β galactosidase assay testing the same pairwise interactions. Bars show calculated Miller units

with the dashed line marking the cutoff from the negative control (bottom). Error bars show standard deviation. (C) Spot titer assay of RN4220 *S. aureus* cells harboring *GpsB^{SA}-GFP mutations (PE355, GGS2, LH17-LH20, LH32, LH35, LH36) and plated on media containing 1 mM IPTG. (D) Production of GpsB^{SA}-GFP (LH141) and *GpsB^{SA}-GFP (LH142-LH144, LH159-LH162) in *S. aureus* RN4220 Δ*spa* cells. Blots were probed with antibody raised against GpsB^{SA}-GFP and a total protein stained gel was used as a loading control.

Figure S3

Superimposition of the crystal structures of *S. pneumoniae (*PDB ID 6GQA), *B. subtilis* (PDB ID 4UG3), *L. monocytogenes* (PDB ID 4UG1), GpsB and the *S. aureus* homology model. (A) Side view. (B) Top view. (C) The equivalent of *S. aureus* D41 is strictly conserved in *S. pneumoniae, B. subtilis, and L. monocytogenes*, where it functions as a hydrogen bond acceptor for three residues. These interactions are an important force that stabilizes this region. The subscripts A and B denote the two protomers in the dimer.

Figure S4

Homology model of *S. aureus* GpsB. The N-terminal LEELR section of α-helix 2 with (a) native and (b) inserted LEE residues colored in yellow.

Figure S5

(A) β galactosidase assay testing pairwise interactions of GpsB^{SA} (LH39-LH40) with TarG (SKB1-SKB2) and TarH (SKB3-SKB4). Bars show calculated Miller units with the dashed line marking the cutoff from the negative control. (B) *S. aureus* cell lysates of GpsB^{SA}-FLAG (LH38)

and GFP-TarG^{SA} (LH136) were combined and subjected to co-immunoprecipitation assay. Load (lysate), flow through, wash 1, wash 2, wash 3, and eluate fractions were subjected to immunoblotting. (C) Purified WTA extracts from strains of SH1000 *S. aureus* cells containing the empty vector (EV; PES5) or a plasmid for *gpsB^{SA}* overexpression (PES13). Cells were grown to mid-log phase and then induced with 1 mM IPTG for 3 h prior before collection and extraction of WTAs. Extracts were analyzed via electrophoresis on a native PAGE gel and visualized through Alcian blue and silver staining. (D) Fluorescence micrographs showing localization of GFP-TarG in RN4220 *S. aureus* cells (LH136) imaged at mid-log phase with no inducer added. Scale bar 1 µm. (E) Measurement of autolysis in strains overproducing GpsB^{SA} (GGS1) and GpsB$^{\Delta LEE}$ (AH2) and an empty vector control (PE355) both with and without treatment of 5 µg/ml targocil. (F) Immunoblot of *E. coli* cell lysates harboring plasmids containing T18-GpsB (LH39) and T18-ΔLEE (LH47) were probed with anti-GpsB antibody. Coomassiee-stained gel of total protein served as a loading control.

Figure S6

Multiple sequence alignments of (A) TagH/TarH and (B) TagG/TarG of *Bacillus subtilis* (Bs), *Staphylococcus aureus* (Sa), *Enterococcus faecalis* (Ef), and *Listeria monocytogenes* (Lm) are shown. The alignment was generated using Clustal Omega. The residues mutated in the extragenic suppressors of *B. subtilis* TagH/TagG shown in Fig. 4A are highlighted.

Table S1

Strains and oligonucleotides used in this study.

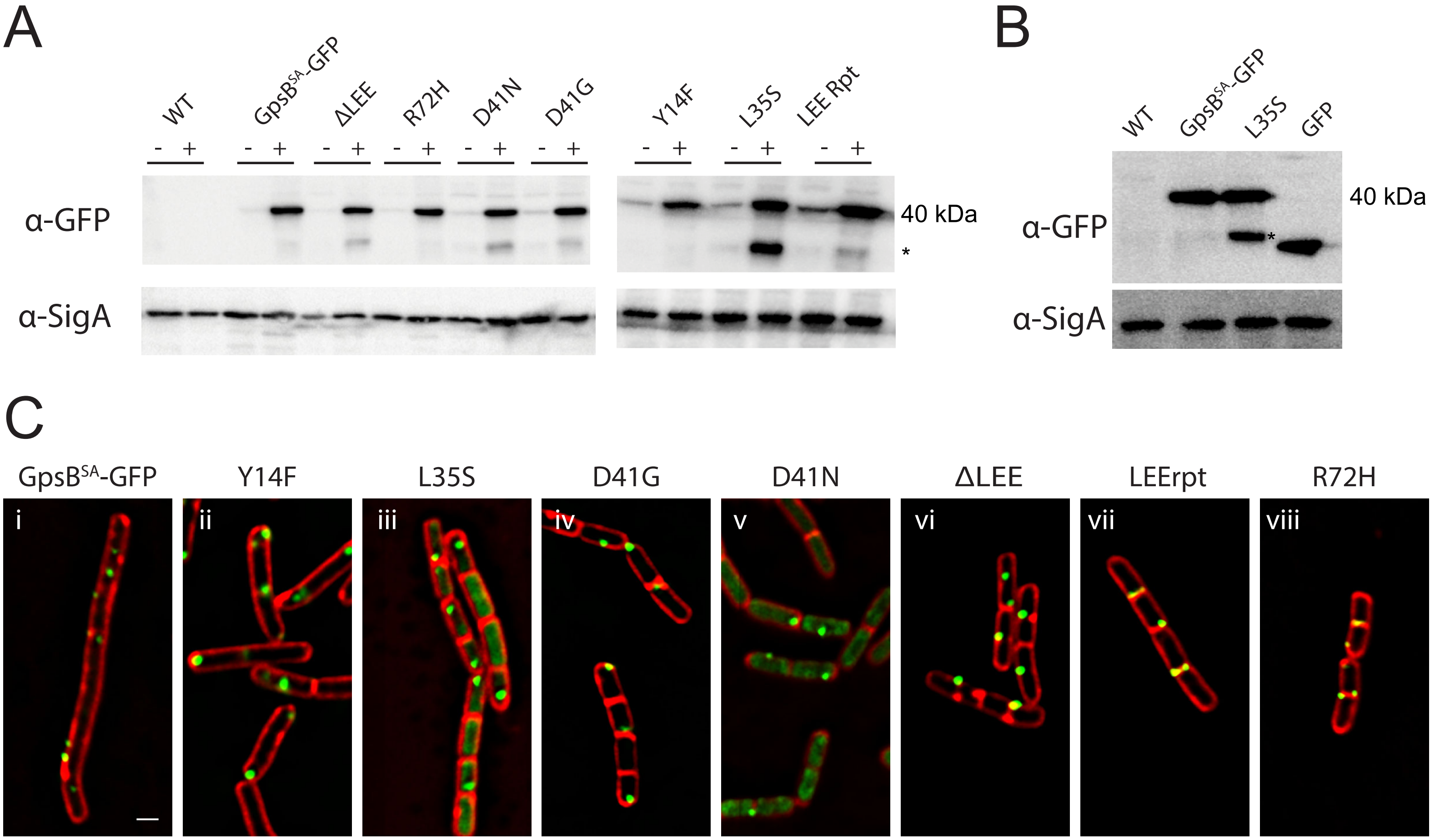
A
WT
GpsB^SA-GFP
ΔLEE
R72H
D41N
D41G
Y14F
L35S
LEE Rpt
- +
α-GFP
40 kDa
*
α-SigA
B
WT
GpsB^SA-GFP
L35S
GFP
40 kDa
α-GFP
*
α-SigA
C
GpsB^SA-GFP
Y14F
L35S
D41G
D41N
ΔLEE
LEErpt
R72H
i
ii
iii
iv
v
vi
vii
viii

Figure S1

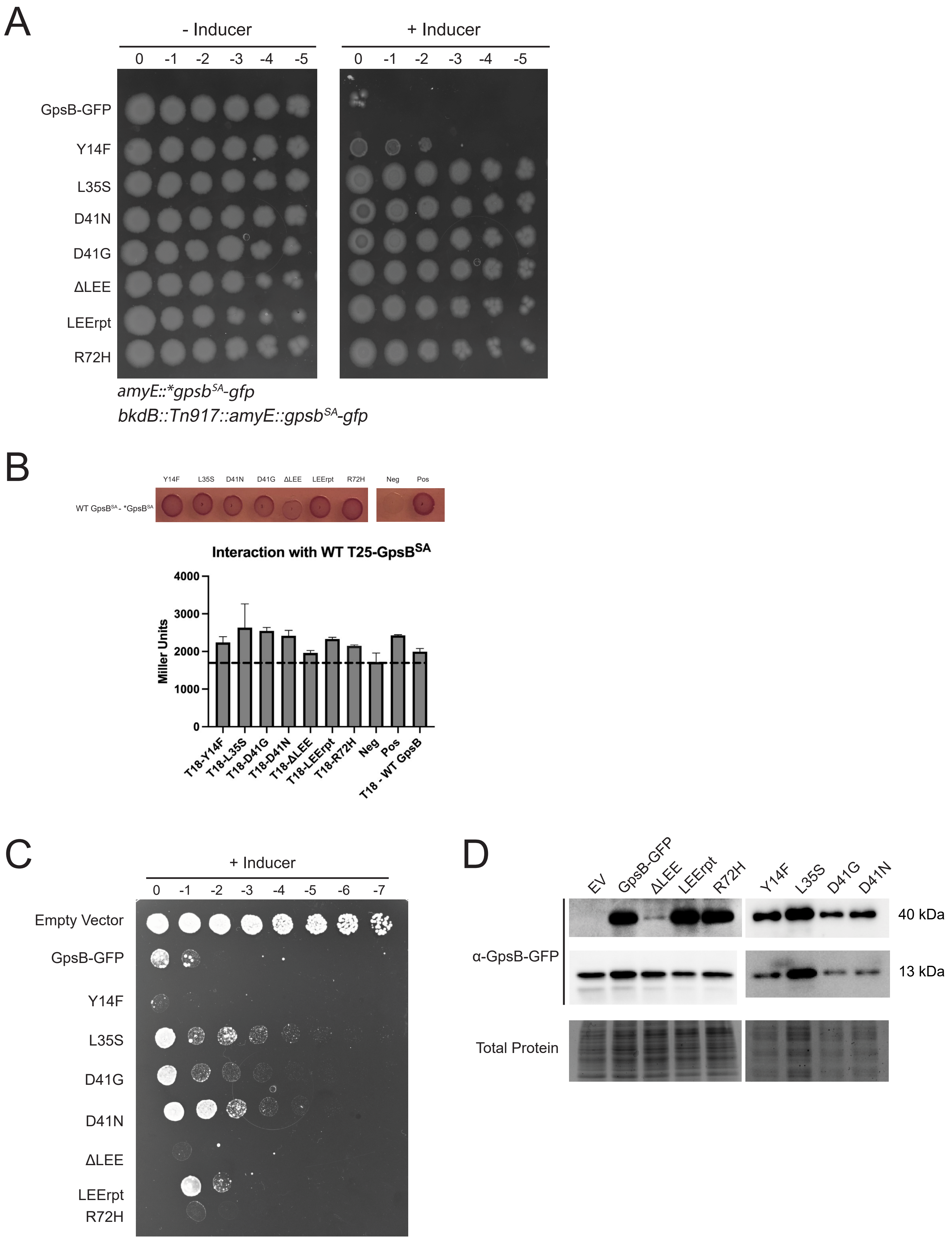
A
- Inducer
+ Inducer
0 -1 -2 -3 -4 -5
GpsB-GFP
Y14F
L35S
D41N
D41G
ΔLEE
LEErpt
R72H
amyE::*gpsb^SA-gfp
bkdB::Tn917::amyE::gpsb^SA-gfp
B
Y14F L35S D41N D41G ΔLEE LEErpt R72H Neg Pos
WT GpsB^SA - *GpsB^SA
Interaction with WT T25-GpsB^SA
Miller Units
0
1000
2000
3000
4000
T18-Y14F
T18-L35S
T18-D41G
T18-D41N
T18-ΔLEE
T18-LEErpt
T18-R72H
Neg
Pos
T18 - WT GpsB
C
+ Inducer
0 -1 -2 -3 -4 -5 -6 -7
Empty Vector
GpsB-GFP
Y14F
L35S
D41G
D41N
ΔLEE
LEErpt
R72H
D
EV
GpsB-GFP
ΔLEE
LEErpt
R72H
Y14F
L35S
D41G
D41N
α-GpsB-GFP
40 kDa
13 kDa
Total Protein

Figure S2

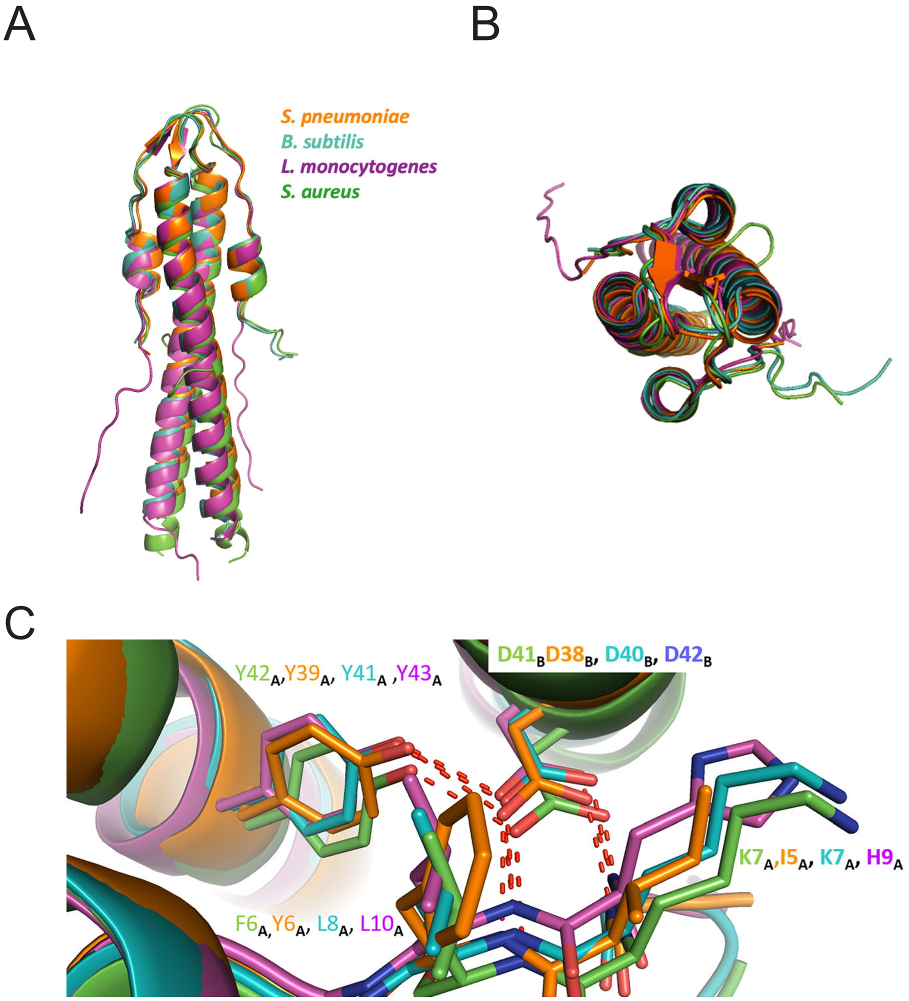
A
B
S. pneumoniae
B. subtilis
L. monocytogenes
S. aureus
C
Y42A, Y39A, Y41A, Y43A
D41B D38B, D40B, D42B
K7A, I5A, K7A, H9A
F6A, Y6A, L8A, L10A

Figure S3

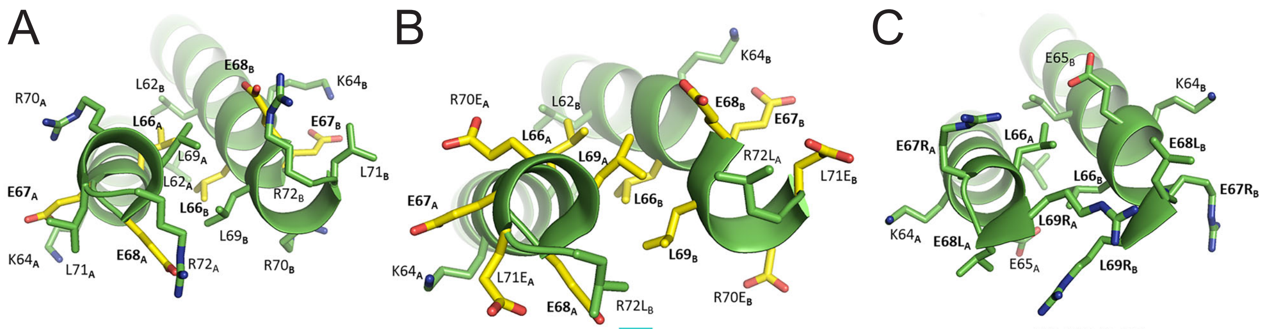

66-LEELR-72

LEE

66-LEELEEL-72

LEE repeat

66-LRLR-69

LEE deletion

Figure S4

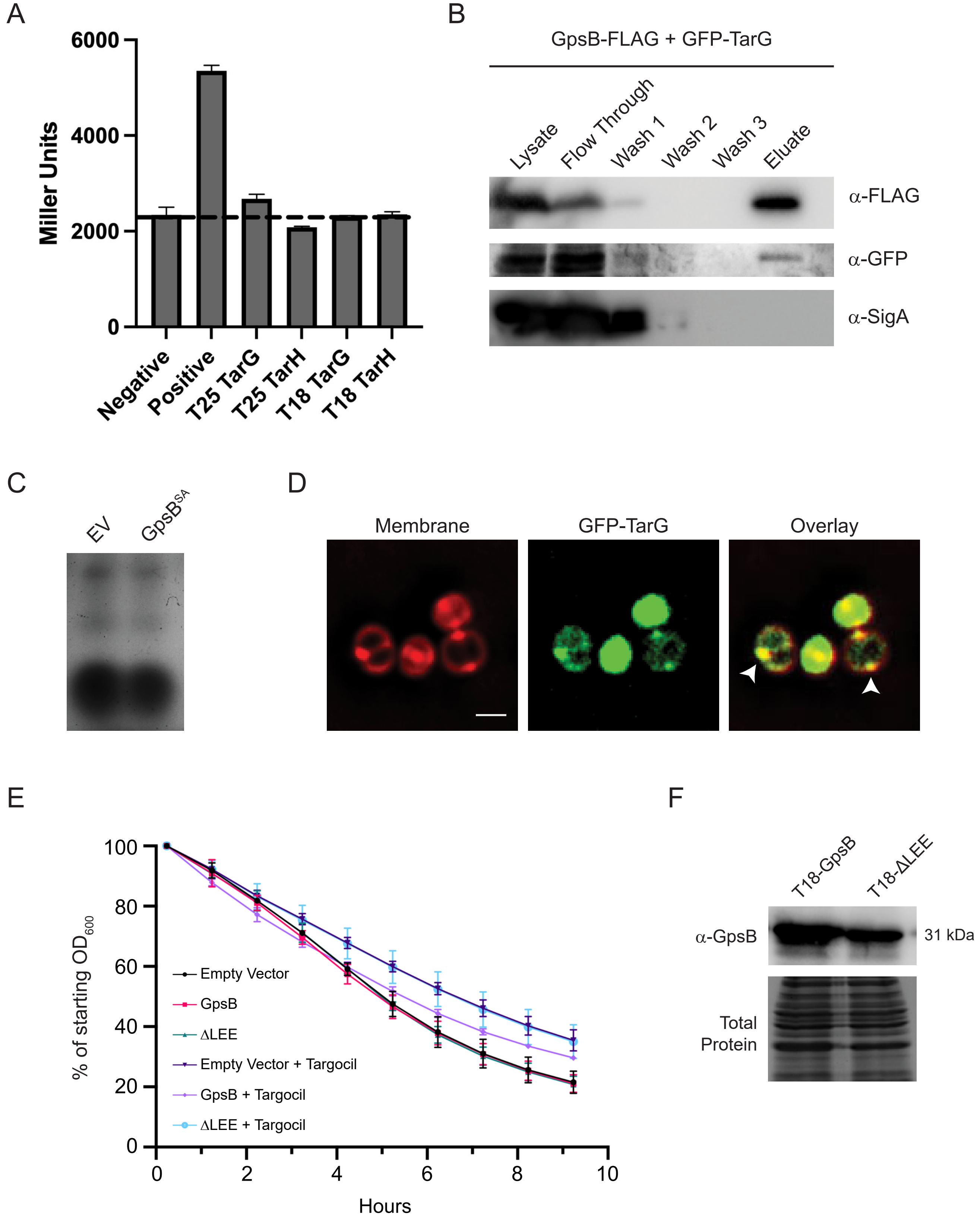
A
Miller Units
6000
4000
2000
0
Negative
Positive
T25 TarG
T25 TarH
T18 TarG
T18 TarH
B
GpsB-FLAG + GFP-TarG
Lysate
Flow Through
Wash 1
Wash 2
Wash 3
Eluate
α-FLAG
α-GFP
α-SigA
C
EV
GpsB^SA
D
Membrane
GFP-TarG
Overlay
E
100
80
60
40
20
0
% of starting OD600
Empty Vector
GpsB
ΔLEE
Empty Vector + Targocil
GpsB + Targocil
ΔLEE + Targocil
0
2
4
6
8
10
Hours
F
T18-GpsB
T18-ΔLEE
α-GpsB
31 kDa
Total Protein

Figure S5

A

```
Bs-TagH      -----MKLKVSFRNVSKQYHLYKKQSDKIKGLFFPAKD--NGFFAVRNVSFDVYEGETIG   53
Sa-TarH      -----MNVSVNIKNVTKEYRIYRTNKERMKDALIPKHKN-KTFFALDDISLKAYEGDVIG   54
Ef-TagH      MKKVEKEIKVRSTLVTKEYDLYKKKSDKVKALFKFYHRNVPRFWALKGVSFEVRAGESVG   60
Lm-TagH      -------MKVSFKHVSKEYDLYQNKSDKIKGLFLPKSKRVQSFWALRNVCFDVYDGETVG   53
                    :.*    *:*:* :*:.:.:::*  :         *:*: .:.:..  *: :*

Bs-TagH      FVGINGSGKSTMSNLLAKIIPPTSGEIEMNGQPSLIAIAAGLNNQLTGRDNVRLKCLMMG   113
Sa-TarH      LVGINGSGKSTLSNIIGGSLSPTVGKVDRNGEVSVIAISAGLSGQLTGIENIEFKMLCMG   114
Ef-TagH      IIGINGSGKSTLSNVISGIIPPTSGTMEVNGDTSIIAIGAGLKYQLTGLENIRLKALMSG   120
Lm-TagH      LIGINGSGKSTISNIMSGVIPPTQGEVVINGETSLIAIAVGLKGPLSGLENIRLKLLMHG   113
             ::*********:**::.  : ** * :  **: *:***..**.  *:* :*:.:* *  *

Bs-TagH      LTNKEIDDMYDSIVEFAEIGDFINQPVKNYSSGMKSRLGFAISVHIDPDILIIDEALSVG   173
Sa-TarH      FKRKEIKAMTPKIIEFSELGEFIYQPVKKYSSGMRAKLGFSINITVNPDILVIDEALSVG   174
Ef-TagH      MTNKEIDKKMDEIVAFADLGDFINQPVKSYSSGMKSRLGFAIAVHNDPDILVIDEALSVG   180
Lm-TagH      LKGSQIDSLLPSIIEFADIGDFINQPIKNYSSGMRSRLGFAISVHTDPDILVVDEALSVG   173
             :. .:*.    .*: *:::*:** **:*.*****:::***:* :  :****::*******

Bs-TagH      DQTFYQKCVDRINEFKKQGKTIFFVSHSIGQIEKMCDRVAWMHYGELRMFDETKTVVKEY   233
Sa-TarH      DQTFAQKCLDKIYEFKEQNKTIFFVSHNLGQVRQFCTKIAWIEGGKLKDYGELDDVLPKY   234
Ef-TagH      DETFYQKCVDKIMEFKESGKTIFFVSHALGQVEKLCDRTIWMHYGDLRMFGPTEEVMAEY   240
Lm-TagH      DQTFYQKCVDKINEFKAQGKTIVFVSHSLGQVKSLCDKIIWMHHGEVREIGDAAEVAEKY   233
             *:** ***:*:* *** ..***.**** :**:..:* :  *:. *.::  .    *  :*

Bs-TagH      KAFIDWFNKLSKKEKETYKKEQTEERKKEDPEAFARFR---------------KKKKKP   277
Sa-TarH      EAFLNDFKKKSKAEQKEFRNKLDESRFVIK------------------------------   264
Ef-TagH      RKFIDWFKKQPKKEQKAYQDKYKENQKAFNLEKLAEKSQDDSLLKEQHLSEQMIQKNLKR   300
Lm-TagH      DEFVKWFNKQPN------------------------------------------------   245
               *:. *:*  :

Bs-TagH      KSLANAIQIAILSILTVFMAGTMFFNAPLRTIASFGAIPQNEVKNHHGDAKGKSEERLTA   337
Sa-TarH      ------------------------------------------------------------   264
Ef-TagH      TPLGEKMTIPTKGLLTLAIVGTLFCSLV-----SFSGRSISYALKHPVDA-------IVQ   348
Lm-TagH      ------------------------------------------------------------   245

Bs-TagH      INKQGFIANEKAAAYKDQGLKQKADVTLPFGTKVTVAAKGKQAAKIKFDGHSYYVKQSAV   397
Sa-TarH      ------------------------------------------------------------   264
Ef-TagH      V-------------FK--------------------------------------------   351
Lm-TagH      ------------------------------------------------------------   245

Bs-TagH      ATNMKHAELHATAFTSYVSQNAASSYEYFLKFLGDSSTSIQSKLNGYTEGNKADGRKTLN   457
Sa-TarH      ------------------------------------------------------------   264
Ef-TagH      ------------------------------------------------------------   351
Lm-TagH      ------------------------------------------------------------   245

Bs-TagH      FDYEKISYVLENDKATELIFHNISPINPASLSLSDSDVLYDSSKKRFLVNTDDQVFAVDN   517
Sa-TarH      ------------------------------------------------------------   264
Ef-TagH      ------------------------------------------------------------   351
Lm-TagH      ------------------------------------------------------------   245

Bs-TagH      EEHTLTLMLK   527
Sa-TarH      ----------   264
Ef-TagH      ----------   351
Lm-TagH      ----------   245
```

B

```
Bs-TagG      MNDLLRILREQITSFPLILRLAAYETKSKYQMNYLGVLWQFLNPLIQMLAYWFVFGMGIR   60
Sa-TarG      MSAIGTVFKEHVKNFYLIQRLAQFQVKIINHSNYLGVAWELINPVMQIMVYWMVFGLGIR   60
Ef-TagG      MKEIAAVIKEQFNHAGIIFRMSRYEDKATYQSHYLGLAWQILNPAIQIGIYYLVFGLGVN   60
Lm-TagG      MKQVMEVIKEQIKNLPMIFRIARYEDKATYQSHYLGLAWQILNPLIQIAIYYFVFGFGMN   60
             *. :  :::*:..   :* *:: :: *   : :***: *:::** :*:  *::***:*:.

Bs-TagG      KGGPVTTGAGEVPFIIWMLAGLIPWFFISPTILDGSNSVFKRINMVAKMNFPISSLPSVA   120
Sa-TarG      SNAP----IHGVPFVYWLLVGISMWFFINQGILEGTKAITQKFNQVSKMNFPLSIIPTYI   116
Ef-TagG      GN----RKVDGVPFIIWMLIGITAWFYINSSVLGASNSIHRQVGMVAKMKFPVSILPSIN   116
Lm-TagG      AK----SGS-DASYIEWMLAGIIPWFFISAVILQGANSIYNKIGMVSKMNFPMSILPNIT   115
                         . :: *:* *:  **:*.  :* .:::: .:.. *:**:**:* :*.

Bs-TagG      IASNLFSYMIMMVIYIIVLLVNGVFPSVHWLQYIYYFICMIAFMFSFSLFNSTISVLIRD   180
Sa-TarG      VTSRFYGHLGLLLLVIIACMFTGIYPSIHIIQLLIYVPFCFFLTASVTLLTSTLGVLVRD   176
Ef-TagG      IISNLSSYIPMVAIVIGSLFLAGVSPTVYWVQFIYYFIAMVIFLFAFGLLNATITVLIRD   176
Lm-TagG      IVSNLTSYFTMMVILLGLLAINGTPITIYWVQYLYYFVAMIAFLFSVTLFNATISVLVRD   175
             : *.: .:: :: : :     . *   ::: :* : *.    . :  :. *:.:*: **:**

Bs-TagG      YQFLLQAVTRLLFFLLPIFWDVNAKLGQSHPELVPVLKLNPLFYIIEGFRNSFLDGAWFF   240
Sa-TarG      TQMLMQAILRILFYFSPILWLPKNHGI--SGLIHEMMKYNPVYFIAESYRAAILYHEWYF   234
Ef-TagG      YHIFLQSVLRLLFYVSGPIWDINNRNM--PEWLIKLLRLNPFYYLIEGFRDAFLSRGWVW   234
Lm-TagG      YYIMLQSVMRVLFYVTGIVWNLET-ML--PQWLVDLLKLNPIYYVVNGFRETFLMNKGFW   232
               :::*:: *:**:.    .*  :          :  ::: **.::: :.:* ::*      :

Bs-TagG      HDM-KYTLYFWLFTFLLLLVGSILHMKFRDKFVDFL   275
Sa-TarG      MDHWKLMLYNFGIVAIFFAIGAYLHMKYRDQFADFL   270
Ef-TagG      EKT-TYTLIFWGMTLTLLIIGSHLHMKFRARFVDYI   269
Lm-TagG      ESP-SYTMYFWLITLTLLFVGATLHMKFRERFVDYL   267
              .  .  :  : :.  :: :*: ****:*  :*.*::
```

Figure S6

Table S1 Strains and oligonucleotides used in this study.

Strains used in this study

Species	Strain	Genotype	Source
B. subtilis	PY79	Wildtype	[1]
B. subtilis	GG8	*amyE::$P_{hyperspank}$-gpsB^{SA}-gfp*	[2]
B. subtilis	PE448	*amyE::$P_{hyperspank}$-*gpsB$^{SA-L35S}$-gfp*	[2]
B. subtilis	PE377	*amyE:: $P_{hyperspank}$-*gpsB$^{SA-LEErpt}$-gfp*	This study
B. subtilis	CS89	*amyE:: $P_{hyperspank}$-*gpsB$^{SA-D41N}$ -gfp*	This study
B. subtilis	CS90	*amyE:: $P_{hyperspank}$-*gpsB$^{SA-\Delta LEE}$ -gfp*	This study
B. subtilis	CS91	*amyE:: $P_{hyperspank}$-*gpsB$^{SA-R72H}$ -gfp*	This study
B. subtilis	CS92	*amyE:: $P_{hyperspank}$-*gpsB$^{SA-Y14F}$ -gfp*	This study
B. subtilis	CS93	*amyE:: $P_{hyperspank}$-*gpsB$^{SA-D41G}$ -gfp*	This study
B. subtilis	LH72	*bkdB::Tn917::amyE::$P_{hyperspank}$-gpsB-gfp (specR)*	This study
B. subtilis	LH73	*bkdB::Tn917::amyE::$P_{hyperspank}$-gpsB-gfp (ermR)*	This study
B. subtilis	LH75	*bkdB::Tn917::amyE::$P_{hyperspank}$-gpsB-gfp amyE::$P_{hyperspank}$-*gpsB$^{SA-L35S}$-gfp*	This study
B. subtilis	LH79	*bkdB::Tn917::amyE::$P_{hyperspank}$-gpsB-gfp amyE:: $P_{hyperspank}$-*gpsB$^{SA-LEErpt}$-gfp*	This study
B. subtilis	LH76	*bkdB::Tn917::amyE::$P_{hyperspank}$-gpsB-gfp amyE:: $P_{hyperspank}$-*gpsB$^{SA-D41N}$ -gfp*	This study
B. subtilis	LH78	*bkdB::Tn917::amyE::$P_{hyperspank}$-gpsB-gfp amyE:: $P_{hyperspank}$-*gpsB$^{SA-\Delta LEE}$ -gfp*	This study
B. subtilis	LH80	*bkdB::Tn917::amyE::$P_{hyperspank}$-gpsB-gfp amyE:: $P_{hyperspank}$-*gpsB$^{SA-R72H}$ -gfp*	This study
B. subtilis	LH74	*bkdB::Tn917::amyE::$P_{hyperspank}$-gpsB-gfp amyE:: $P_{hyperspank}$-*gpsB$^{SA-Y14F}$ -gfp*	This study
B. subtilis	LH77	*bkdB::Tn917::amyE::$P_{hyperspank}$-gpsB-gfp amyE:: $P_{hyperspank}$-*gpsB$^{SA-D41G}$ -gfp*	This study
B. subtilis	RL4709	*amyE::$P_{hyperspank}$-gfp*	(R. Losick Lab)
B. subtilis	SK15	*lacA::Pxyl-dcas9 amyE::Pveg-sgRNA (tagG)*	This study; derived from BEC35710 (^BGSC)
B. subtilis	SK16	*lacA::Pxyl-dcas9 amyE::Pveg-sgRNA (tagH)*	This study; derived from BEC35700 (^BGSC)
B. subtilis	SK17	*lacA::Pxyl-dcas9 amyE::Pveg-sgRNA (tagG)*; *bkdB::Tn917::amyE::$P_{hyperspank}$-gpsB-gfp*	This study
B. subtilis	SK18	*lacA::Pxyl-dcas9 amyE::Pveg-sgRNA (tagH)*; *bkdB::Tn917::amyE::$P_{hyperspank}$-gpsB-gfp*	This study
B. subtilis	PE528	*tagH::P_{xyl}-gfp-tagH (1-648)*	This study; derived from [3]
B. subtilis	GG19	*amyE::$P_{hyperspank}$-gpsB^{BS}-gfp*	[2]
S. aureus	SH1000	Wildtype	[4]
S. aureus	RN4220	Wildtype	Lab stock
S. aureus	SEJ1	RN4220 Δ*spa*	[5]
S. aureus	PES5	SH1000 with pCL15 Empty Vector	[2]
S. aureus	PE355	RN4220 with pCL15 Empty Vector	[2]
S. aureus	PES6	SH1000 pCL15 backbone *P_{spac}-gpsB^{SA}-gfp*	[2]
S. aureus	GGS2	RN4220 pCL15 backbone *P_{spac}-gpsB^{SA}-gfp*	[2]
S. aureus	PES13	SH1000 pCL15 backbone *P_{spac}-gpsB^{SA}*	[2]
S. aureus	GGS1	RN4220 pCL15 backbone *P_{spac}-gpsB^{SA}*	[2]
S. aureus	LH36	RN4220 pCL15 backbone *P_{spac}-*gpsB$^{SA\ L35S}$-gfp*	[2]
S. aureus	LH35	RN4220 pCL15 backbone *P_{spac}-*gpsB$^{SA\ LEErpt}$-gfp*	This study
S. aureus	LH19	RN4220 pCL15 backbone *P_{spac}-*gpsB$^{SA\ D41N}$-gfp*	This study
S. aureus	LH17	RN4220 pCL15 backbone *P_{spac}-*gpsB$^{SA\ \Delta LEE}$-gfp*	This study
S. aureus	LH18	RN4220 pCL15 backbone *P_{spac}-*gpsB$^{SA\ R72H}$-gfp*	This study
S. aureus	LH32	RN4220 pCL15 backbone *P_{spac}-*gpsB$^{SA\ Y14F}$-gfp*	This study
S. aureus	LH20	RN4220 pCL15 backbone *P_{spac}-*gpsB$^{SA\ D41G}$-gfp*	This study
S. aureus	LH136	RN4220 pJB67 backbone *P_{cad}-gfp-tarG*	This study
S. aureus	AH2	RN4220 pCL15 backbone *P_{spac}-*gpsB$^{SA\ \Delta LEE}$*	This study
S. aureus	LH140	RN4220 with pCL15 Empty Vector	This study
S. aureus	LH141	RN4220 Δspa pCL15 backbone *P_{spac}-gpsB^{SA}-gfp*	This study
S. aureus	LH162	RN4220 Δspa pCL15 backbone *P_{spac}-*gpsB$^{SA\ L35S}$-gfp*	This study
S. aureus	LH144	RN4220 Δspa pCL15 backbone *P_{spac}-*gpsB$^{SA\ LEErpt}$-gfp*	This study
S. aureus	LH160	RN4220 Δspa pCL15 backbone *P_{spac}-*gpsB$^{SA\ D41N}$-gfp*	This study
S. aureus	LH142	RN4220 Δspa pCL15 backbone *P_{spac}-*gpsB$^{SA\ \Delta LEE}$-gfp*	This study

S. aureus	LH143	RN4220 Δspa pCL15 backbone P_{spac}-*$gpsB^{SA\ R72H}$-*gfp*	This study
S. aureus	LH161	RN4220 Δspa pCL15 backbone P_{spac}-*$gpsB^{SA\ Y14F}$-*gfp*	This study
S. aureus	LH159	RN4220 Δspa pCL15 backbone P_{spac}-*$gpsB^{SA\ D41G}$-*gfp*	This study
E. coli	BTH101	*Adenylate cyclase deficient reporter strain for BACTH; F', cya-99, araD139, galE15, galK16, rpsL1 (Str^R), hsdR2, mcrA1, mcrB1, relA1*	[6]
E. coli	LH39	T18-linker $gpsB^{SA}$	This study
E. coli	LH40	T25-linker $gpsB^{SA}$	This study
E. coli	LH43	T18-linker *$gpsB^{SA\ LEErpt}$	This study
E. coli	LH44	T25-linker *$gpsB^{SA\ LEErpt}$	This study
E. coli	LH45	T18-linker *$gpsB^{SA\ L35S}$	This study
E. coli	LH46	T25-linker *$gpsB^{SA\ L35S}$	This study
E. coli	LH47	T18-linker *$gpsB^{SA\ \Delta LEE}$	This study
E. coli	LH48	T25-linker *$gpsB^{SA\ \Delta LEE}$	This study
E. coli	LH49	T18-linker *$gpsB^{SA\ Y14F}$	This study
E. coli	LH50	T25-linker *$gpsB^{SA\ Y14F}$	This study
E. coli	LH51	T18-linker *$gpsB^{SA\ D41G}$	This study
E. coli	LH52	T25-linker *$gpsB^{SA\ D41G}$	This study
E. coli	LH53	T18-linker *$gpsB^{SA\ D41N}$	This study
E. coli	LH54	T25-linker *$gpsB^{SA\ D41N}$	This study
E. coli	LH55	T18-linker *$gpsB^{SA\ R72H}$	This study
E. coli	LH56	T25-linker *$gpsB^{SA\ R72H}$	This study
E. coli	SKB3	T18-linker *tarH*	This study
E. coli	SKB4	T25-linker *tarH*	This study
E. coli	SKB1	T18-linker *tarG*	This study
E. coli	SKB2	T25-linker *tarG*	This study
E. coli	PE87	pUT25-zip	[6]
E. coli	PE88	pUT18-zip	[6]
E. coli	PE84	pEB355	[6]
E. coli	PE83	pEB354	[6]
E. coli	pCL15	pCL15	[7]
E. coli	pLH5	pCL15 backbone P_{spac}-*$gpsB^{SA\ \Delta LEE}$-*gfp*	This study
E. coli	pLH6	pCL15 backbone P_{spac}-*$gpsB^{SA\ R72H}$-*gfp*	This study
E. coli	pLH7	pCL15 backbone P_{spac}-*$gpsB^{SA\ D41N}$-*gfp*	This study
E. coli	pLH8	pCL15 backbone P_{spac}-*$gpsB^{SA\ D41G}$-*gfp*	This study
E. coli	pLH13	pCL15 backbone P_{spac}-*$gpsB^{SA\ Y14F}$-*gfp*	This study
E. coli	pPE78	pCL15 backbone P_{spac}-*$gpsB^{SA\ LEErpt}$-*gfp*	This study
E. coli	pPE80	pCL15 backbone P_{spac}-*$gpsB^{SA\ L35S}$-*gfp*	This study
E. coli	pPE46	pCL15 backbone P_{spac}-$gpsB^{SA}$-*gfp*	[2]
E. coli	pPE45	pCL15 backbone P_{spac}-$gpsB^{SA}$	[2]
E. coli	pLH64	pJB67 backbone P_{cad}-*gfp-tarG*	This study
E. coli	pAH1	pCL15 backbone P_{spac}-*$gpsB^{SA\ \Delta LEE}$	This study

*denotes mutant $gpsB^{SA}$
^BGSC - Bacillus Genetic Stock Center

Oligonucleotides used in this study

Primer	Sequence (5' to 3')
oP36	AAAAAGCTTACATAAGGAGGAACTACTATGTCAGATGTTTCATTGAAATTATCAGCA
oP38	AAAGCATGCTTATTTACCAAATACAGCTTTTTCTAAGTTTGA
oGG2	AAAGTCGACTTATTTGTATAGTTCATCCATGCC
BTH11	AATAAGAATTCATGTCAGATGTTTCATTGAAATTATCAGC
BTH12	GCTGTATTTGGTAAATAACTCGAGTTATT
BTH61	AATAAGAATTCATGAACGTTTCGGTAAACATTAAAAATG
BTH62	AATAACTCGAGTTATTTAATAACGAAGCGGGACTCATCG
BTH63	AATAAGAATTCATGTCAGCAATAGGAACAG
BTH64	AATAACTCGAGTTACAAGAAGTCTGCAAATTGATCTC
oLH11	AATAAGTCGACACATAAGGAGGAACTACTATGAGTAAAGGAGAAGAACTTTTCAC
oLH12	AATAAGGATCCTTTGTATAGTTCATCCATGCC
oLH13	AATAAGGATCCATGTCAGCAATAGGAACAG
oLH14	AATAAGAATTCTTACAAGAAGTCTGCAAATTG

References

1. Youngman P, Perkins JB, Losick R. Construction of a cloning site near one end of Tn917 into which foreign DNA may be inserted without affecting transposition in Bacillus subtilis or expression of the transposon-borne erm gene. Plasmid. 1984;12(1):1-9. Epub 1984/07/01. doi: 10.1016/0147-619x(84)90061-1. PubMed PMID: 6093169.
2. Eswara PJ, Brzozowski RS, Viola MG, Graham G, Spanoudis C, Trebino C, et al. An essential Staphylococcus aureus cell division protein directly regulates FtsZ dynamics. Elife. 2018;7. Epub 2018/10/03. doi: 10.7554/eLife.38856. PubMed PMID: 30277210; PubMed Central PMCID: PMCPMC6168285.
3. Formstone A, Carballido-Lopez R, Noirot P, Errington J, Scheffers DJ. Localization and interactions of teichoic acid synthetic enzymes in Bacillus subtilis. J Bacteriol. 2008;190(5):1812-21. Epub 2007/12/25. doi: 10.1128/JB.01394-07. PubMed PMID: 18156271; PubMed Central PMCID: PMCPMC2258661.
4. Horsburgh MJ, Aish JL, White IJ, Shaw L, Lithgow JK, Foster SJ. sigmaB modulates virulence determinant expression and stress resistance: characterization of a functional rsbU strain derived from Staphylococcus aureus 8325-4. J Bacteriol. 2002;184(19):5457-67. Epub 2002/09/10. doi: 10.1128/JB.184.19.5457-5467.2002. PubMed PMID: 12218034; PubMed Central PMCID: PMCPMC135357.
5. Yu W, Missiakas D, Schneewind O. Septal secretion of protein A in Staphylococcus aureus requires SecA and lipoteichoic acid synthesis. Elife. 2018;7. Epub 2018/05/15. doi: 10.7554/eLife.34092. PubMed PMID: 29757141; PubMed Central PMCID: PMCPMC5962339.
6. Battesti A, Bouveret E. The bacterial two-hybrid system based on adenylate cyclase reconstitution in Escherichia coli. Methods. 2012;58(4):325-34. Epub 2012/07/31. doi: 10.1016/j.ymeth.2012.07.018. PubMed PMID: 22841567.
7. Luong TT, Lee CY. The arl locus positively regulates Staphylococcus aureus type 5 capsule via an mgrA-dependent pathway. Microbiology (Reading). 2006;152(Pt 10):3123-31. Epub 2006/09/29. doi: 10.1099/mic.0.29177-0. PubMed PMID: 17005991.
